# Supplementary material for: The effectiveness of anti-stigma interventions for reducing mental health stigma in young people: A systematic review and meta-analysis
Source: Glob Ment Health (Camb). 2023 Jul 10;10:e39. doi: 10.1017/gmh.2023.34 (PMC10579682; doi:10.1017/gmh.2023.34)
Supplement: Supplementary file 1 [file S2054425123000341sup.zip › S2054425123000341sup002.docx]

**Appendix 2 Risk Assessment**

Risk of Bias 2 for Randomised Controlled Trials

|  | Domain 1  Randomisation Process | Domain 2  Deviations from the intended interventions | Domain 3  Missing outcome data | Domain 4  Measurement of the outcome | Domain 5  Selection of the reported result | Overall |
| --- | --- | --- | --- | --- | --- | --- |
| Vila-Badia et al. (2012) | Some concerns | Some concerns | Low | Some concerns | Some concerns | High |
| Townsend et al. (2019) | Some concerns | Some concerns | Low | Some concerns | Some concerns | High |
| O’Mara et al. (2013) | Some concerns | Some concerns | Some concerns | Some concerns | Some concerns | High |
| Nguyen et al. (2020) | Some concerns | Some concerns | Some concerns | Some concerns | Some concerns | High |
| Gonçalves et al. (2015) | Some concerns | Some concerns | Some concerns | Some concerns | Some concerns | High |
| Winkler et al. (2017) | Some concerns | Some concerns | Some concerns | Some concerns | Some concerns | High |
| Economou et al. (2011) | Some concerns | Some concerns | Some concerns | Some concerns | Some concerns | High |
| Mulfinger et al. (2018) | Low | Some concerns | Low | Some concerns | Some concerns | Some concerns |
| Cheetham et al. (2020) | Some concerns | Some concerns | Low | Some concerns | Some concerns | High |
| Economou et al. (2014) | Some concerns | Some concerns | Low | Some concerns | Some concerns | High |
| Milin et al. (2018) | Low | Some concerns low | Some concerns | Some concerns | Some concerns | High |
| Howard et al. (2018) | Low | Some concerns | Low | Some concerns | Some concerns | Some concerns |
| Staniland & Byrne (2013) | High | Some concerns low | Low | Some concerns | Some concerns | High |
| Cangas et al. (2017) | Some concerns | Some concerns | Low | Some concerns | Some concerns | High |
| Painter et al. (2017) | Some concerns | Some concerns | Some concerns | Some concerns | Some concerns | High |
| Saporito et al. (2013) | Some concerns | Some concerns | Low | Some concerns | Some concerns | High |

Risk of Bias 2 for Cluster-Randomised Trials

|  | Domain 1 | | Domain 2  Deviations from the intended interventions | Domain 3  Missing outcome data | Domain 4  Measurement of the outcome | Domain 5  Selection of the reported result | Overall |
| --- | --- | --- | --- | --- | --- | --- | --- |
|  | Randomisation Process | Timing of identification or recruitment of participants in a cluster-randomized trial |  |  |  |  |  |
| Pinto-Foltz et al. (2011) | Some concerns | Low | Low | Low | Low | Low | Some concerns |
| Perry et al. (2014) | Low | Low | Some concerns | Some concerns | Low | Some concerns | Some concerns |
| Link et al. (2020) | Low | Low | Some concerns | Some concerns | Low | Some concerns | High |
| DeLuca (2020) | Low | Low | Some concerns | Low | Low | Low | Some concerns |
| Chisholm et al. (2016) | Low | Low | Some concerns | High | Low | Some concerns | High |
| Ahmad et al. (2020) | Some concerns | Low | Some concerns | High | Low | Some concerns | High |
