# Supplementary figures and images for: The effectiveness of anti-stigma interventions for reducing mental health stigma in young people: A systematic review and meta-analysis
Source: Glob Ment Health (Camb). 2023 Jul 10;10:e39. doi: 10.1017/gmh.2023.34 (PMC10579682; doi:10.1017/gmh.2023.34)

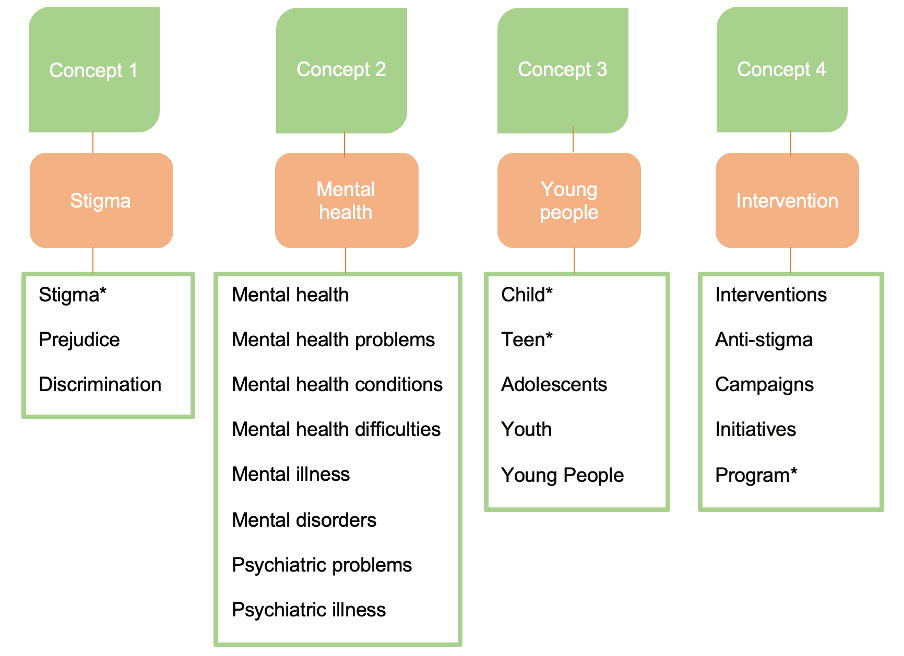

Supplement: Supplementary file 1 [file S2054425123000341sup.zip › S2054425123000341sup001.jpg]

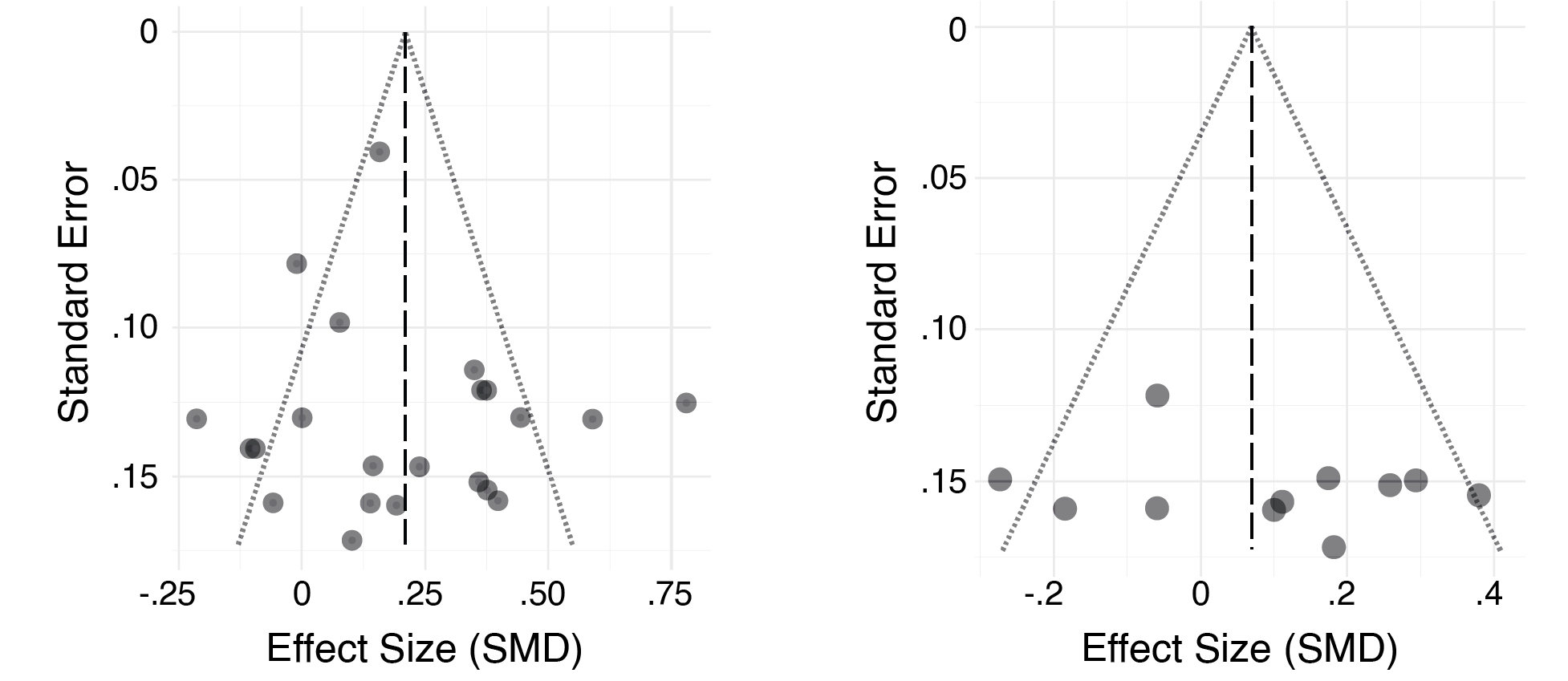

Supplement: Supplementary file 1 [file S2054425123000341sup.zip › S2054425123000341sup003.jpg]

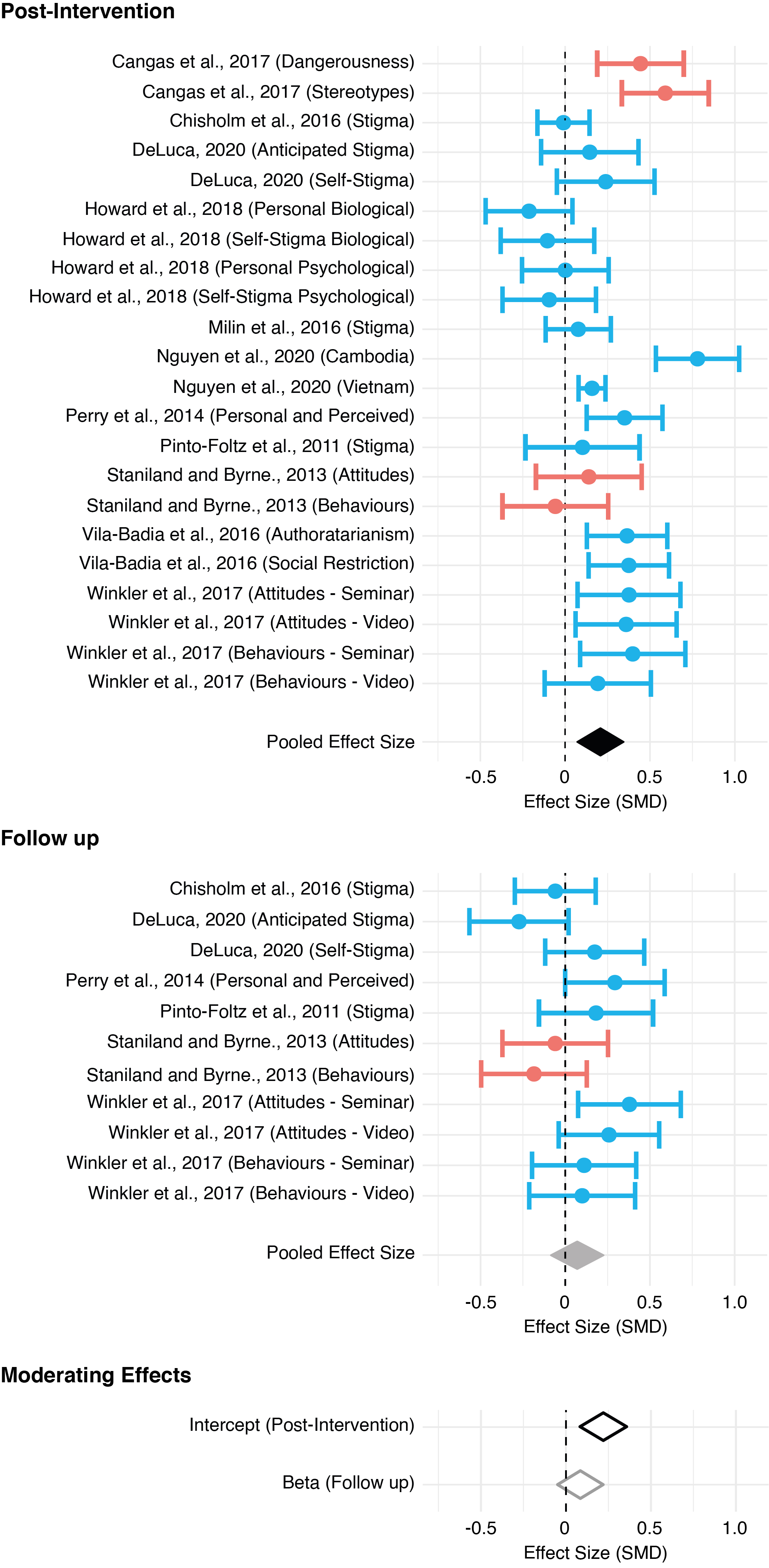

Supplement: Supplementary file 1 [file S2054425123000341sup.zip › S2054425123000341sup004.jpg]
